# Correlation between tunability and anisotropy in magnetoelectric voltage tunable inductor (VTI)

**DOI:** 10.1038/s41598-017-14455-0

**Published:** 2017-11-22

**Authors:** Yongke Yan, Liwei D. Geng, Lujie Zhang, Xiangyu Gao, Sreenivasulu Gollapudi, Hyun-Cheol Song, Shuxiang Dong, Mohan Sanghadasa, Khai Ngo, Yu U. Wang, Shashank Priya

**Affiliations:** 1 Center for Energy Harvesting Materials and Systems, Virginia Tech, Blacksburg, VA 24061 USA; 2 Department of Materials Science and Engineering, Michigan Technological University, Houghton, MI 49931 USA; 3 Center for Power Electronics Systems (CPES), Virginia Tech, Blacksburg, VA 24061 USA; 4 School of Engineering, Peking University, Beijing, 10084 China; 5 Center for electronic materials, Korea Institute of Science and Technology (KIST), Seoul, 02792 Republic of Korea; 6 Weapons Development and Integration Directorate, Aviation and Missile Research, Development, and Engineering Center, US Army RDECOM, Redstone Arsenal, AL 35898 USA

## Abstract

Electric field modulation of magnetic properties via magnetoelectric coupling in composite materials is of fundamental and technological importance for realizing tunable energy efficient electronics. Here we provide foundational analysis on magnetoelectric voltage tunable inductor (VTI) that exhibits extremely large inductance tunability of up to 1150% under moderate electric fields. This field dependence of inductance arises from the change of permeability, which correlates with the stress dependence of magnetic anisotropy. Through combination of analytical models that were validated by experimental results, comprehensive understanding of various anisotropies on the tunability of VTI is provided. Results indicate that inclusion of magnetic materials with low magnetocrystalline anisotropy is one of the most effective ways to achieve high VTI tunability. This study opens pathway towards design of tunable circuit components that exhibit field-dependent electronic behavior.

## Introduction

Control of magnetic properties by electric field (abbreviated as *E*-field = voltage/thickness) is of fundamental and technological importance for realizing lighter, smaller, and efficient power electronics through overall reduction in the number of passives^1,2^. Passive components with added-tunability are being sought to provide adaptability to different circuit conditions. They will add a major leap in size optimization, controllability, and circuit design strategies. Multiferroic magnetoelectric composites comprising of magnetostrictive and piezoelectric phases take advantage of strain-mediated interaction that allows control of magnetic permeability through *E*-field^1,3,4^. This control can be exploited to re-design one of the fundamental components for electronic circuits, inductor, that is extensively used in control circuits, power converters, filters, etc. *E*-field or voltage tunable inductors (VTIs) represent a new class of magnetoelectric components that can have significant effect on enhancing the efficiency of power electronics and reducing the number of passives by actively changing the magnitude of inductance depending upon the operating conditions. For example – major challenge with conventional point-of-load (POL) converter is achieving high efficiency and high power density due to the lack of high performance inductor. Large number of output capacitors are required due to slow current slew rate limited by large inductance, which is used to achieve low ripple and high efficiency. In a tunable inductor based POL converter, a large inductance is realized at steady state while a smaller inductance is achieved at load transient to increase current slew rate, and hence, reduce the number of capacitors by a significant amount. In contrast to conventional magnetic components or devices, where tunability is achieved either by mechanical tuning through bias magnet(s) or by adjusting the electric current in solenoids/electromagnets, VTIs exhibit smaller footprint, larger tunability and lower energy consumption.

**Figure 1:**
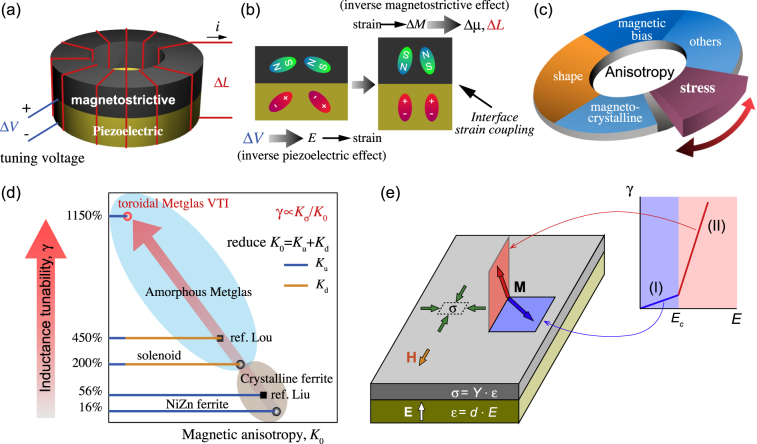
Voltage tunable inductor (VTI). Schematic of (**a**) the structure of VTI. (**b**) The working principle of VTI. VTI operates on the principle of modulation of magnetic properties in the magnetostrictive layer through strain generated at the interface by applying voltage to the piezoelectric layer. The magnitude of change in the magnetic property in the magnetostrictive layer is dependent upon various anisotropic factors. (**c**) Pie diagram of the various types of anisotropy (magnetocrystalline anisotropy *K*_*u*_, shape anisotropy *K*_*d*_, stress induced anisotropy for magnetostrictive materials ${K}_{\sigma }$, and others including magnetic bias, exchange and random anisotropy) influencing the tunability of permeability and inductance. (**d**) State-of-the-art inductance tunability of VTIs. Under condition of stress induced anisotropy being dominant, small magnetocrystalline and shape anisotropy are essential requirements towards achieving large inductance tunability. The magnetic anisotropy factor *K*_0_ represents the summation of initial magnetocrystalline and shape anisotropy. By minimizing *K*_0_ this study was able to significantly advance the magnitude of tunability. Sphere shape data points represent this study, and square shape data points refer to prior studies (Ref. Lou^5^ and Ref. Liu^6^). (**e**) Magnetization **M** behavior under different magnitude of tuning voltage *E* or stress $\sigma$: low field Regime I (blue) corresponds to rotation of in-plane magnetization and high-field Regime II (red) corresponds to rotation of out-of-plane magnetization.

Figure 1a schematically illustrates the structure of VTI based upon strain-mediated magnetoelectric effect, implying change in the magnetic property with applied *E*-field. In this figure, there are two constituent phases in inductor core, a magnetostrictive phase and a ferroelectric phase, which are coupled through strain transfer at interface. Figure 1b depicts the working principle of VTI. The tuning voltage, *V*, induces strain in the piezoelectric phase due to the inverse piezoelectric effect. The strain is mechanically transferred to the magnetostrictive phase, inducing domain reorientation or a magnetization change (Δ*M*) through the inverse magnetostrictive effect or Villari effect^7^. The magnetization change results in the permeability change (Δ*μ*), which is the principle parameter governing the magnitude of inductance change (Δ*L*). Based on this principle, several VTI architectures have been explored in literature.

Lou *et al*.^5^ have reported electrically tunable solenoid inductors with a multiferroic core consisting of two layers of amorphous magnetic ribbons (Metglas 2605) and one Pb(Zr,Ti)O_3_ (PZT) slab which exhibited a large tunable inductance range of 450%, ~3 times enhanced peak quality factor, and extended operation frequency. The change of inductance and quality factor was due to a strong magnetoelectric coupling in the multiferroic composite core, which led to electric field induced permeability change (as reflected in the magnetic hysteresis loops measured by vibration sample magnetometer (VSM)). Liu *et al*.^6^ have demonstrated an electrically tunable ME inductor comprising of a ring-type PZT/MnZn-ferrite/PZT laminate composite. The ring shape ferrite core eliminates the demagnetization field and leads to high permeability and high inductance. This VTI exhibited an inductance tunability of up to 56.6% in a wide range of frequency.

Prior studies indicate that the magnitude of tunability is strongly dependent on the composition of magnetic materials and shape/structure of magnetoelectric composite core^5,6,8–10^. Fundamentally, it is very important to understand and distinguish the significance of each factor affecting the magnitude of inductance tunability in VTI. We found that the inductance tunability strongly correlates with underlying anisotropies in magnetic material (magnetocrystalline anisotropy, shape anisotropy, stress induced anisotropy and magnetic field bias induced anisotropy) as shown in Fig. 1c. Our investigations demonstrate that small magnetocrystalline anisotropy and shape anisotropy are essential requirements towards achieving large inductance tunability when stress induced anisotropy plays dominant role (Fig. 1c). By minimizing magnetic anisotropy *K*_0_ (representing the summation of initial magnetocrystalline anisotropy and shape anisotropy), this study demonstrates large magnitude of inductance tunability γ as shown in Fig. 1d. Further, we experimentally show that the tunability of Metglas ME inductor has two linear regimes separated by the critical electric field *E*_c_ (low field regime I: *E* < *E*_c_, and high field regime II: *E* > *E*_c_). We provide an analytical model, revealing that two regimes of tunability behaviors are related to two different magnetization rotation processes (regime I: in-plane rotation, and regime II: out-of-plane rotation) as shown in Fig. 1e. This foundational understanding should advance the development and implementation of VTIs. The fabrication processes used for VTIs are shown in Supplementary Fig. S1 and discussed in the experimental section.

## Results

**Figure 2:**
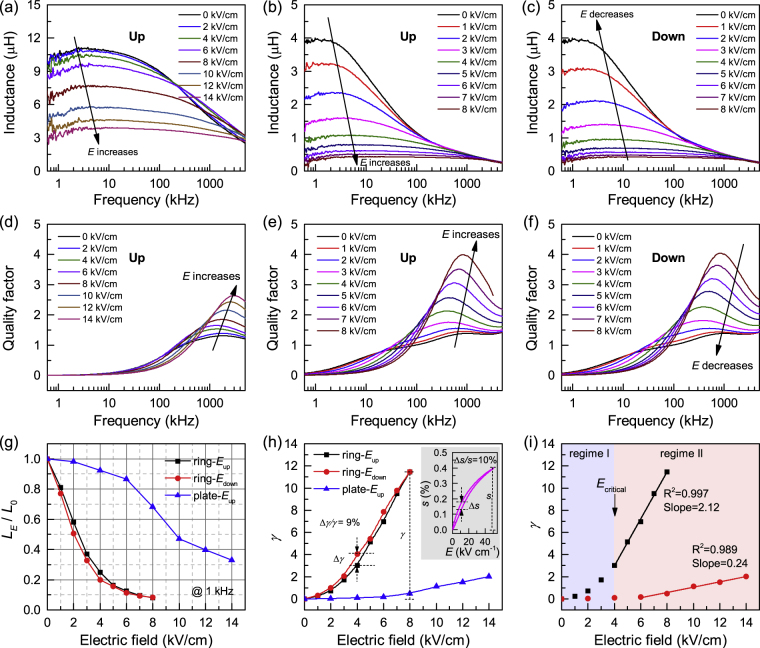
Tunability of Metglas VTI. (**a**)(**b**)(**c**) Inductance and (**d**)(**e**)(**f**) quality factor spectra of the plate-type (*E*-field up), ring-type (*E*-field up), and ring-type (*E*-field down) of Metglas/PMN-PZT voltage tunable inductor, respectively. The electric field dependence of (**g**) *L*_E_/*L*_0_, (**h**) tunability *γ* of Metglas/PMN-PZT plate-type and ring-type VTI at 1 kHz. (**i**) linear fitting of *γ* - *E for* ring-type voltage tunable inductor. Giant changes in the magnitude of inductance can be seen with slight increase in the applied electric field. The changes are reversible as shown by the drive in both up and down directions. Small hysteresis in the up and down drive is related to the non-linearity in the piezoelectric material. Ring-type inductor shows rapid drop in inductance value with applied electric field. Tunability of ring-type inductor reaches up to 1150% as compared to 46% for the plate-type demonstrating the important role of shape anisotropy.

Figure 2a–c show the inductance spectra of Metglas/PMN-PZT VTI under different applied DC electric fields. The inductance of ring-type ME inductor decreases more rapidly than that of plate-type ME inductor with increase in applied electric field on piezoelectric layer. For both plate-type and ring-type inductors, the inductance rolls off quickly at higher frequencies. This behavior is closely related to the large eddy current loss in Metglas foils at high frequency. Eddy currents are closed loops of electrical current within inductor core produced in response to external ac magnetic field, following Faraday’s law of induction. The eddy current creates a magnetic field that opposes any change in the external magnetic field, thereby, reducing the net magnetic flux and decreasing the magnet inductance. It should be noted here the decrease of inductance at the low frequency could be due to the parasitic resistance [Supplementary Figure S2] and capacitance caused by the measuring wire connected to the inductor. Further, the noise from materials, environment and electrical instrumentation have stronger influence at low frequencies. Figure 2d–f show the quality factor spectra of the ME inductors under different electric fields. The quality factor (*Q*) is defined as the ratio of the energy stored in a component to the energy dissipated by the component, given as $Q=\pi \frac{{E}_{store}}{{E}_{dissipated}}=\omega \frac{L}{R}$, where *L* is the series inductance, *R* is the series resistance, and ω is the angular frequency. In general, the *Q*-factor increases at low frequency, then reaches its maximum (*Q*_max_) and after that it starts decaying. At low frequency, *Q* increases with increase in frequency ω, while at high frequency *Q* will decrease with the decrease in *L* or increase in *R* [Supplementary Figure S2], especially as one approaches the resonance frequency. The peak quality factors of both plate-type and ring-type ME inductors are improved with the increase in the applied control electric field. The reason for such improvement of quality factors is due to the decrease of permeability (*μ*_r_) and hence increased skin depth $(\delta \propto \sqrt{1/{\mu }_{r}})$, reduced resistance *R* and eddy current loss. To clearly show the effect of applied electric field on the inductance of different types of inductors, *L*_E_/*L*_0_ as a function of electric field is plotted in Fig. 2g, where *L*_E_ and *L*_0_ are the inductance with and without electric field. It can be seen that the inductance of ring-type inductor decreases much faster than plate-type inductor. In order to quantify the tunability of inductance with applied control electric field, the inductance tunability *γ* is defined as $\gamma =({L}_{0}-{L}_{E})/{L}_{E}$. As shown in Fig. 2h, the *γ* of ring-type ME inductor at 1 kHz reaches up to 1150% under the electric field of 8 kV cm^−1^, which is much higher than 46% for plate-type ME inductor under same electric field. Here, it can be observed that the tunability of this ME inductor has about 9% hysteresis (${\rm{\Delta }}\gamma /\gamma$) when the *E*-field was rising (*E*-field up) and falling (*E*-field down). This behavior should be related to the strain hysteresis of PMN-PZT piezoelectric ceramic as shown in the inset of Fig. 2h.

The inductance change of VTI is closely related to the strain mediated magnetoelectric coupling within the core, which leads to electric field induced change of the permeability of the Metglas. The relative permeability *μ*_r_ of the magnetic materials can be expressed as^11^:

1 $${\mu }_{r}=\chi +1=\frac{{\mu }_{0}{M}_{s}^{2}}{2|{K}_{0}+{K}_{\sigma }|}+1,$$

where

2 $${K}_{\sigma }=\frac{3}{2}{\lambda }_{s}\sigma ,$$

where *μ*_0_ is the vacuum permeability, *M*_s_ is the saturation magnetization, *K*_0_ is the initial magnetic anisotropy (including magnetocrystalline anisotropy and shape anisotropy), *K*_σ_ is the stress-induced magnetoelastic anisotropy (stress anisotropy), *λ*_s_ is the saturation magnetostriction coefficient, and *σ* is applied stress. In ME inductor, the stress applied on Metglas is imposed by the piezoelectric layer. Therefore, *μ*_r_ can be further correlated with electric field applied on piezoelectric phase, and the relationship can be given as:

3 $${\mu }_{r}=\frac{{\mu }_{0}{M}_{s}^{2}}{2{K}_{0}+3{\lambda }_{s}Y{d}_{eff}E}+1,$$

where *d*_eff_ is effective piezoelectric strain coefficient, *Y* is the Young’s modulus and *E* is applied electric field on piezoelectric layer. Since the physical dimension change of inductor is very small (<0.5%, due to piezoelectric effect and magnetostriction effect) and the ${\mu }_{r}$ of magnetic materials is significantly larger than 1, the change of inductance should be directly proportional to *μ*_r_. Then the relation between tunability *γ* and electric field *E* can be derived as:

4 $$\gamma =\frac{{L}_{0}-{L}_{E}}{{L}_{E}}=\frac{3}{2}\cdot \frac{{\lambda }_{s}}{{K}_{0}}\cdot Y\cdot {d}_{eff}\cdot E.$$

From Fig. 2i, it can be observed that *γ* varies with *E* linearly when *E* > 4 kV cm^−1^, as predicted by Equations (4). The slope ratios of *γ* - *E* plot for ring-type and plate-type inductors is 2.12 and 0.24, respectively, a difference of 8.9 times. This indicates that the ring-type VTI has much higher sensitivity to the applied electric field than plate-type ME inductor. Considering the ${\lambda }_{s}$, and *Y* of magnetic materials and the *d*_eff_ of piezoelectric materials are intrinsic properties of the materials, the difference in *γ* - *E* slope should be related to the difference of initial magnetic anisotropy ${K}_{0}$ between ring-type Metglas and plate-type Metglas. It is well-known that the shape of magnetic material can induce magnetic anisotropy, the so-called shape anisotropy, due to the formation of demagnetization field when the magnetic materials are exposed to the magnetic field. Since the magnetocrystalline anisotropy is the same for both ring-type and plate-type Metglas inductors, the difference of initial magnetic anisotropy ${K}_{0}$ between ring-type Metglas and plate-type Metglas should be related to the difference of shape anisotropy, i.e., absence of demagnetization field in ring-type Metglas but the presence of demagnetization field in plate-type Metglas.

**Figure 3:**
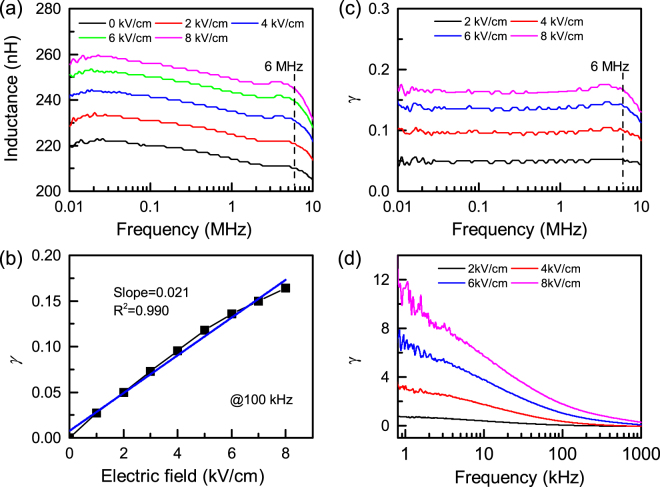
Tunability of ferrite VTI. (**a**) Inductance spectra of ring-type ferrite/PMN-PZT VTI. (**b**) Tunability *γ* of ring-type ferrite/PMN-PZT VTI under different electric field at 100 kHz. Frequency dependence of tunability of (**c**) NiZnCu-ferrite/PMN-PZT and (**d**) Metglas/PMN-PZT VTI. A significant contrast in the frequency dependence of ferrite vs. Metglas based composites can be noticed. Metglas based composites exhibit rapid decay in tunability with frequency while the ferrite based composites have almost flat response until MHz range.

Figure 3a shows the measured inductance spectra of ring-type ferrite/PMN-PZT inductor under different applied DC electric fields. The roll-off frequency of NiZnCu-ferrite ring-type inductor is up to 6 MHz, which is much higher than ~3 kHz of Metglas ring-type inductor. Figure 3b shows calculated tunability under different applied DC electric field. The tunability of this ring-type ferrite inductor is 16% under 8 kV cm^−1^ which is ~72 times smaller than 1150% achieved in the Metglas ring-type inductor. The slope of tunability vs electric field in ring-type ferrite ME inductor is about 0.021, which is 100 times smaller than 2.12 observed in the ring-type Metglas inductor (Fig. 2i).

Let’s consider the limiting factor of tunability based on Equation (4). The saturation magnetostriction coefficient ${\lambda }_{s}$ for NiZnCu-ferrite and Metglas are 19 ppm and 27 ppm, respectively^12^. The Young’s modulus *Y* for NiZnCu-ferrite and Metglas are about 120 GPa and 110 GPa, respectively^12,13^. The effective piezoelectric strain coefficient *d*_eff_ for NiZnCu-ferrite and Metglas inductors are the same because of the same piezoelectric materials (PMN-PZT) used. Therefore, the large difference of *γ* - *E* slope between NiZnCu-ferrite and Metglas inductors should also be related to the difference of initial magnetic anisotropy ${K}_{0}$. In contrast to the difference of magnetic anisotropy ${K}_{0}$ between ring-type Metglas and plate-type Metglas, there is no difference in the shape anisotropy in both ring-type NiZnCu-ferrite and ring-type Metglas inductors because there is no demagnetization field in both cases (closed magnetic flux circuit forms in the ring-type inductors). Therefore, the 100 times difference of initial magnetic anisotropy ${K}_{0}$ is mainly related to the difference of intrinsic magnetocrystalline anisotropy of crystalline NiZnCu-ferrite and amorphous Metglas, which is well consistent with the reported *K* value of 38 J m^−3^ for Metglas (ref.^11^) and 1000 J m^−3^ to 6300 kJ m^−3^ for most Ni ferrites and Mn ferrites (depending on the doping element and stoichiometry of spinel structure^14,15^).

## Discussion

Table 1 lists the reported inductance or permeability tunability of VTI. It can be seen that the magnitude of tunability is strongly dependent on the composition of magnetic materials and shape/structure of magnetoelectric composite core. Therefore, it is very important to fundamentally understand and distinguish the significance of each factor on the tunability of inductance in ME inductor. Since electric manipulation of magnetization is achieved via the magnetoelectric coupling in magnetostrictive/piezoelectric composite, the tunability will be strongly related to: (1) the stress sensitivity or tunability of inductance or permeability in magnetic phase, which further correlate with anisotropy; (2) strain coupling between piezoelectric phase and magnetic phase; and (3) the piezoelectric strain coefficient of piezoelectric phase. Here, we will discuss these three aspects.

**Table 1:** Inductance or permeability tunability of different ME inductors.

Materials	Shape	Tunability	Field	Frequency	Ref.
Metglas/PZT	plate-plate	450%	12 kV cm^−1^	0.1 kHz	5
Metglas/PZT	plate-plate (unimorph)	~240%	5 kV cm^−1^	1 kHz	18
Metglas/PZT	plate-plate (bimorph)	750%	5 kV cm^−1^	1 kHz	18
MnZn ferrite/PZT	ring-ring	56%	3 kV cm^−1^	72 kHz	6
MnZn ferrite/PZT	ring-bar	20%	5 kV cm^−1^	15 kHz	10
Bi_0.7_Dy_0.3_FeO_3_	ring (single phase)	3.5%	0.65 kV cm^−1^	1000 kHz	8
Metglas/PMN-PZT	plate-plate	200%	12 kV cm^−1^	1 kHz	this study
Metglas/PMN-PZT	ring-ring	1150%	8 kV cm^-1^	1 kHz	this study
NiZnCu-Ferrite/PMN-PZT	ring-ring	16%	8 kV cm^−1^	6000 kHz	this study

### Correlation between inductance tunability and anisotropy

Since the change of physical dimension of ME tunable inductors is negligible, the inductance tunability is strongly related to the change of permeability of magnetic materials. Permeability is the degree of magnetization that a material obtains in response to an applied magnetic field. For ferro- and ferri-magnetic materials, this process is often described by the flux density *B* or magnetization *M* vs. magnetic field *H* curves. The ratio of *B* to *H* is called the permeability, $\mu$. One of the most important factors which strongly affects the shape of the *B*-*H* or *M*-*H* curve is the magnetic anisotropy. There are several kinds of anisotropy: magnetocrystalline anisotropy, shape anisotropy, stress induced anisotropy for magnetostrictive materials and magnetic field induced anisotropy. Each of these anisotropies (either intrinsic or extrinsic) may become predominant in specific circumstances. From Table 1, the variation of inductance can be mainly classified according to the materials composition, shape and structure, which can be further related to the magnetocrystalline anisotropy *K*_*u*_, shape anisotropy *K*_*d*_ due to demagnetization effect, and stress induced anisotropy ${K}_{\sigma }$. To investigate the effect of external applied bias magnetic field, which is very important for application because the working point of inductor is not always zero, a bias field induced anisotropy *K*_*H*_ is also considered in this study. Basically, to achieve high tunability in ME inductor, the contribution of magnetoelastic stress induced anisotropy ${K}_{\sigma }\,\,$should be significant or dominant with respect to the total anisotropy of material. In the following, the correlation of tunability and different anisotropies will be analyzed.

**Figure 4:**
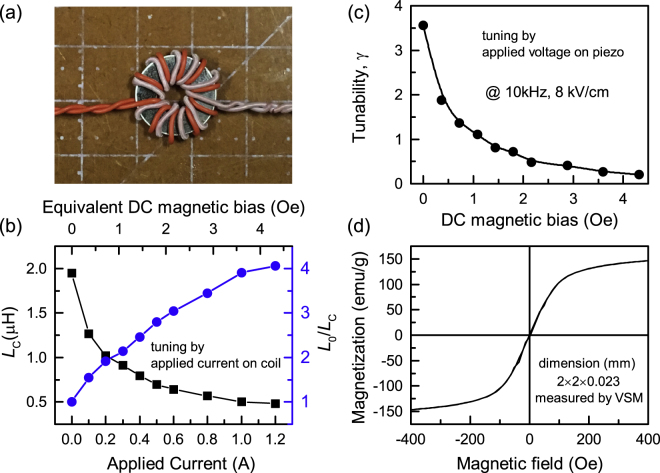
Bias-dependent tunability of Metglas VTI. (**a**) Picture of Metglas/PMN-PZT inductor core with measuring and secondary coil (for applying DC magnetic field). (**b**) Inductance (*L*_C_) and tuning ratio (*L*_0_/*L*_C_) of Metglas/PMN-PZT ME inductor tuned by applying current in secondary coil. (**c**) Tunability *γ* of Metglas/PZT ME inductor tuned by electric field (8 kV cm^−1^) on piezoelectric layer under different DC magnetic bias. (**d**) Magnetization as a function of magnetic field.
- *Stress Induced Anisotropy:* As mentioned above, the tunability of ME inductor is significantly dependent on the percentage of stress induced anisotropy in the total anisotropy. The stress induced anisotropy can be estimated by: ${K}_{\sigma }=\frac{3}{2}{\lambda }_{s}\sigma =\frac{3}{2}{\lambda }_{s}Y{d}_{31}E$. The ${\lambda }_{s}$ of Metglas and NiZnCu ferrite are 27 ppm and 19 ppm, respectively^12^. The *Y* of Metglas and NiZnCu ferrite are 110 GPa and 120 GPa, respectively^12,13^, and *d*_31_ of piezoelectric ceramic PMN-PZT is 420 pC N^−1^ ^17^. Under electric field of *E* = 8 kV cm^−1^, the stress will be 37 MPa and 40 MPa, respectively. ${K}_{\sigma }$ in Metglas and NiZnCu ferrite are 1497 J m^−3^ and 1149 J m^−3^, respectively. The magnetocrystalline anisotropy *K*_*u*_ of Metglas and Ni ferrite are 38 J m^−3^ and 6200 J m^−3^ ^11,16^. Therefore, for ring-type/toroid inductor (without considering shape anisotropy), the tunability of Metglas and NiZnCu ferrite can be expected to be 3940% and 18.5%, respectively. The magnitude of expected tunability have similar trends as observed from the experimental value (1150% and 16% for Metglas and NiZnCu-ferrite ME inductor, respectively). The relatively large difference for Metglas inductor is mainly due to the fast frequency rolling down behavior of inductance and tunability as shown in Figs 2b and 3d, respectively. This result clearly demonstrates that the search for magnetic materials with low magnetocrystalline anisotropy is one of the most effective ways to achieve high inductor tunability.

- *Magnetocrystalline Anisotropy*: The magnetocrystalline anisotropy is termed as the energy required for rotating the magnetization from the easy direction. In this study, two different magnetic materials, namely, amorphous Metglas and crystalline NiZnCu-ferrite, were used to investigate the effect of intrinsic magnetic anisotropy on the tunability of ME inductor. The magnetocrystalline anisotropy *K*_*u*_ of Metglas is 38 J m^−3^ (ref.^11^), and for most Ni- and Mn-ferrites *K*_*u*_ is in the range of 1000 J m^−3^ to 6300 J m^−3^ depending on the doping element and stoichiometry of spinel structure^14–16^. The large difference in *K*_*u*_ leads to the significant difference of tunability between Metglas inductor and ferrite inductor as experimentally observed in this study. Thus, it can be concluded that low magnetocrystalline anisotropy *K*_*u*_ is required to achieve high tunability in ME inductor.
- *Shape Anisotropy*: Depending upon the shape of the inductor, the magnetic circuit (the region occupied by magnetic flux lines) can be open (the flux passes partially through “nonmagnetic” materials, usually air) or closed. In open circuit, a pair of magnetic poles are formed and a demagnetizing field *H*_d_ will be formed in the opposite direction to the magnetization *M*. This shape/demagnetization anisotropy *K*_*d*_ partially balances the stress induced anisotropy ${K}_{\sigma }\,\,$in the total free anisotropy, thereby, reducing the stress sensitivity. In this study, the dimension of Metglas is 14.80 × 2.80 mm × 0.023 mm, the dimensional ratio of long axis to short axis is about 650, *M*_s_ is 146 emu g^−1^, density is 7.18 g cm^−3^ ^12^, the demagnetization factor *N*_1_ is about 1 × 10^−3^ based on the theoretical calculation for rectangular prisms. According to the equation ${K}_{{\rm{d}}1}=\frac{1}{2}{\mu }_{0}{N}_{1}{M}_{s}^{2}$, the *K*_d1_ is about 700 J m^−3^, which is about 18 times the magnetocrystalline anisotropy *K*_*u*_ (38 J m^−3^) of Metglas^11^. The addition of shape anisotropy *K*_*d*_ in plate-type Metglas significantly reduces the tunability of Metglas/PMN-PZT ME inductor.
- *Magnetic Field Induced Anisotropy:* The DC magnetic bias changes the working point of inductor and thereby changes the permeability. Here a second coil was wrapped around the inductor core and the magnetic bias was induced by the current in this second coil as shown in Fig. 4a. Figure 4b shows the inductance (*L*_C_) change and corresponding tuning ratio (*L*_0_/*L*_C_) of Metglas/PMN-PZT ME inductor under different current (DC magnetic bias). With increased current in the coil (equivalently increased DC magnetic bias), the inductance drops rapidly at the beginning and then gradually becomes stable where magnetization reaches saturation. Under different current or equivalent DC magnetic bias, the ME tunability under 8 kV cm^−1^ was measured and shown in Fig. 4c. It can be found that ME tunability *γ* decreases rapidly with increase in the applied magnetic bias due to magnetic bias induced magnetic anisotropy, which can be estimated by: $H=\frac{2{K}_{u}}{{M}_{s}}$. The *M*_s_ is about 146 emu g^−1^ as shown in Fig. 4d. The induced anisotropy by the magnetic bias of 3.6 Oe is about 189 J m^−3^, which is about 5 times of magnetocrystalline anisotropy. Correspondingly, the tunability of ME inductor decreases from 360% to 26%. It should be mentioned here that the additional coil windings affect the measured inductance because two windings are wrapped in a common core (acting as transformer), but will not change the inductance tuning behavior/trend. Further, it also should be noted here that the saturation field in Fig. 4d is much larger than the value in Fig. 4b due to demagnetization field arising from the VSM sample shape and dimension.

### Strain from piezoelectric phase

The electric field (*E*) induced strain ($\varepsilon$) from piezoelectric phase can be given by $\varepsilon ={d}_{eff}E$, where *d*_*eff*_ is effective piezoelectric coefficient. The magnitude of *d*_*eff*_ of piezoelectric phase is not only dependent on the composition (such as PZT, PMN-PT) and microstructure (single crystal, polycrystalline ceramic), but also on the structure (such as unimorph, bimorph) and vibration mode (such as 31-mode, 33-mode). In regard to structure, the *d*_*eff*_ of piezoelectric bimorph is $3/2{d}_{31}\,$due to bending effect, thereby, the strain produced by bimorph structure is 1.5 times that of a single piezoelectric plate (unimorph) under an identical applied electric field. Liu *et al*.^18^ compared the magnetic susceptibility tunability of ferromagnetic amorphous alloy ribbon stressed by a piezoelectric bimorph actuator and a piezoelectric unimorph actuator. The permeability tunability of ME composites reached up to 750% under 5 kV cm^−1^ at 1 kHz for bimorph structure, which is triple that of unimorph structure^18^.

In this study, a new type of piezoelectric material, <001> textured Pb(Mg_1/3_Nb_2/3_)O_3_-Pb(Zr, Ti)O_3_ ceramics is employed, in which the piezoelectric properties (*d*_33_ = 1150 pC N^−1^, *d*_31_ = −420 pC N^−1^, *k*_33_ = 0.88, *k*_31_ = 0.63, and *k*_p_ = 0.84)^17^ are much higher than that of traditional PZT ceramics (for example, PZT-5H: *d*_33_ = 583 pC N^−1^, *d*_31_ = −262 pC N^−1^, *k*_33_ = 0.75, *k*_31_ = 0.38, and *k*_p_ = 0.64). The origin of high piezoelectric response of <001> textured piezoelectric ceramics is due to engineered domain configuration which is in analogous to single crystal^17,19^. Besides the material, the magnitude and distribution of stress also are influenced by the shape of piezoelectric layer. Supplementary Figure S3 shows the impedance spectra of textured PMN-PZT piezoelectric ceramics with different shapes (plate and disc). It can be found that the electromechanical coupling factor of disc shape (*k*_p_ = 0.85) samples is higher than that of plate shape (*k*_31_ = 0.63) sample.

### Stress transfer from the piezoelectric phase to magnetic phase

In the strain-coupled ME composites, both the electric field manipulation of magnetic properties and magnetic field control of electric polarization are directly related to the effectiveness of elastic coupling at the interface of the two phases. The widely used method for synthesis of laminate composites is bonding of magnetostrictive layer using epoxy resin. This method limits the misfit strain at the interface arising due to thermal expansion mismatch between the layers and atomic inter-diffusion and/or chemical reaction between the layers. However, the epoxy layer is much softer than both magnetostrictive alloy and ferroelectric ceramic and thus it will dampen the generated strain resulting in loss of efficiency. In contrast to epoxy bonding, co-firing of layered ME composite can achieve the effective bonding without additional layer; it also provides compatibility with current industrial production process commonly used for fabrication of multilayer capacitors (MLCs), which provides the possibility of cost-effective mass production. Figure 2b shows the co-fired ME inductor made from textured PMN-PZT and NiZnCu-ferrite.

### Frequency limitation

From Table 1 and Fig. 3c,d, it can be seen that the frequency range of inductor is strongly dependent on the materials. Based on the Snoek’s law for unmagnetized materials, the resonance frequency is given by ${\omega }_{r}^{min}{\mu }_{i}^{^{\prime} }\approx \frac{2}{3}\gamma 4\pi {M}_{s}$ ^20^. The materials with high initial permeability ${\mu }_{i}^{\prime}$ has low ferromagnetic resonance frequency (cut-off frequency). Based on the discussion above, the ME inductor with high tunability should have low magnetocrystalline anisotropy. Low magnetocrystalline anisotropy will give high permeability and low cut-off frequency. Metglas has extremely large tunability, but it has narrow frequency range and cannot be used at high frequency. Taking into account the stress factor, the resonance frequency of magnetic materials is given as: ${\omega }_{r}=\gamma {H}_{K}=\gamma \frac{4|{K}_{0}+\sigma {\lambda }_{s}|}{3{M}_{s}}$. Therefore, the stress can also be used to shift the ferromagnetic resonance frequency or cut-off frequency, as clearly evidenced in Fig. 2d,f.

### Two-regime tuning behavior of Metglas VTI

To better analyze the voltage tuning behaviors as show in Fig. 2i, a theoretical model was provided (see Supplementary Information for model details). The model revealed that a transition between the two regimes occurs at stress ${\sigma }_{c}$ given by Supplementary Equation (S5):

5 $${\sigma }_{c}=\frac{2({K}_{u}+{K}_{d3})}{3{\lambda }_{s}}$$

where ${K}_{u}\,\,$is the magnetocrystalline anisotropy, ${\lambda }_{s}\,\,$is the saturation magnetostriction constant, and ${K}_{d3}$ is the shape anisotropy energy along ribbon thickness that depends on the detailed domain microstructures. Such a critical stress determined by Equation (5) corresponds to the critical electric field ${E}_{c}$:

6 $${E}_{c}=\frac{2({K}_{u}+{K}_{d3})(1-\nu )}{3{\lambda }_{s}Y{d}_{31}}$$

which gives *E*_c_ = 2.6 kV cm^−1^ for ring-type inductor and *E*_c_ = 5.6 kV cm^−1^ for plate-type inductor. Here, $\nu$ is the Poisson’s ratio of Metglas.

**Figure 5:**
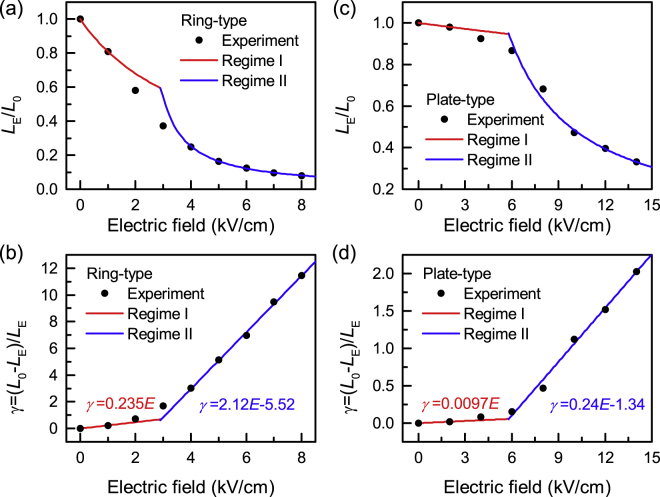
Comparison of theory and experiment. The theory is fitted to reproduce linear tunability of Regime I (red line) and Regime II (blue line) of experimental data (black circles). Deviation of experimental data from theory is attributed to non-uniformities in magnetic domains and material microstructures.

Using extracted values of the relevant parameters, the theoretical predictions of Regime I and II for both ring-type and plate-type inductors are plotted and compared with the experimental data in Fig. 5. These values agree well with the experimental data. At Regime I, magnetizations still align along the material easy-axis due to low electric field, so that the in-plane rotation of magnetizations accounts for the existence of tunability plateau. At Regime II, the electric field is high enough to overcome the material anisotropy to eventually form out-of-plane magnetizations, and the out-of-plane rotation of magnetization results in the linear fast-varying tunability. It is worth noting that the experimental data exhibits a smooth transition rather than a sharp transition between the two regimes, where the tunability gradually deviates from the initial low-field linear segment of Regime I and smoothly approaches the high-field linear segment of Regime II. Such deviation from idealized theoretical model is attributed to the non-uniformities in the layered magnetoelectric inductors, such as spatial variations in *K*_d3_ and $\sigma \,\,$due to the microstructures of magnetic domains in Metglas ribbons and polarization domains in PMN-PZT layer.

In summary, an extremely large inductance tunability (up to 1150% at 1 kHz under 8 kV cm^−1^) was demonstrated in a toroid voltage tunable inductor with a core of Metglas/Pb(Mg_1/3_Nb_2/3_)O_3_-Pb(Zr,Ti)O_3_/Metglas laminated composite. This induction tuning arises from the change of permeability, which further correlates with the change of magnetic anisotropy induced by mechanical stress from the piezoelectric phase. The effects of magnetocrystalline anisotropy, shape anisotropy, stress induced anisotropy, and magnetic field bias induced anisotropy on the tunability of the ME inductor were discussed. It was found that small magnetocrystalline anisotropy and low shape anisotropy are essential requirements to achieve large inductance tunability where stress induced anisotropy is dominant. Further, it was experimentally and theoretically found that the tunability of Metglas ME inductor has two linear regimes separated by the critical electric field *E*_c_ or critical stress *E*_c_ (low field regime: *E* < *E*_c_, and high field regime: *E* > *E*_c_). When subjected to low compressive strain or under low electric field, the easy magnetization axis of the amorphous alloy ribbon still lies within the plane and the magnetization is produced by in-plane rotation process, which accounts for the existence of the plateau.

## Methods

### Sample preparation

Two different processes were used to fabricate VTIs as shown in Supplementary Figure S1:

- *Metglas VTI:* Metglas VTI comprises of magnetic amorphous alloy Metglas bonded with sintered piezoelectric ceramics using epoxy as shown in Supplementary Figure S1a. In Supplementary Figure S1a, the subfigure (i) shows the schematic of inductor core. It consists of one layer of textured PMN-PZT piezoelectric ceramic (500 μm in thickness, poled along thickness direction before bonding with Metglas) and two layers of magnetic Metglas 2605SA1 (each 23 μm). The piezoelectric ceramics have two shapes: long plate and ring disc, as shown in subfigure (ii). Metglas magnetic layers were bonded together with piezoelectric PMN-PZT ceramics using epoxy as shown in subfigure (iii, iv). After bonding, the working coils were wound around the core as shown in subfigure (v).
- *Co-fired ferrite VTI:* Co-fired ferrite VTI was fabricated using low-temperature ferrite/PMN-PZT ceramic composite as show in Supplementary Figure S1b. Textured PMN-PZT (subfigure ii) and NiZnCu-ferrite (subfigure iii) ceramic green tapes were stacked, laminated, and then punched into the ring with inner diameter (ID) of 8 mm and out diameter (OD) of 18 mm. Next, the green samples were co-fired at 930 °C for 3 hours. Subfigures (iv,v) show the ME inductor core and toroidal inductor, respectively. The details in preparation of textured PMN-PZT and NiZnCu-ferrite ceramic green tapes can be found elsewhere^17,21^.

### Characterization

The inductance and quality factor of the inductors were measured by precision impedance analyzer (*L*_s_-*Q* measurement of HP 4194 A). An electric field from 0 to 12 kV cm^−1^ was applied on PMN-PZT piezoelectric ceramic. The magnetization of magnetic materials was measured by a vibration sample magnetometer (VSM-VersaLab, Quantum Design).

## Electronic supplementary material

Supplementary Information
