# Supplementary material for: Correlation between tunability and anisotropy in magnetoelectric voltage tunable inductor (VTI)
Source: Sci Rep. 2017 Nov 22;7:16008. doi: 10.1038/s41598-017-14455-0 (PMC5700207; doi:10.1038/s41598-017-14455-0)
Supplement: Supplementary file 1 — Supplementary Information [file 41598_2017_14455_MOESM1_ESM.pdf]

Correlation between tunability and anisotropy in magnetoelectric voltage tunable inductor (VTI)

Yongke Yan[1], Liwei D. Geng[2], Lujie Zhang[3], Xiangyu Gao[1,4], Sreenivasulu Gollapudi[1], Hyun-Cheol Song[1,5], Shuxiang Dong[1,4], Mohan Sanghadasa[6], Khai Ngo[3], Yu U. Wang[2], and Shashank Priya[1]

[1]Center for Energy Harvesting Materials and Systems, Virginia Tech, Blacksburg, VA 24061, USA

[2]Department of Materials Science and Engineering, Michigan Technological University, Houghton, MI 49931, USA

[3]Center for Power Electronics Systems (CPES), Virginia Tech, Blacksburg, VA 24061, USA

[4]School of Engineering, Peking University, Beijing 10084, China

[5]Center for electronic materials, Korea Institute of Science and Technology (KIST), Seoul 02792, Republic of Korea

[6]Weapons Development and Integration Directorate, Aviation and Missile Research, Development, and Engineering Center, US Army RDECOM, Redstone Arsenal, AL 35898, USA

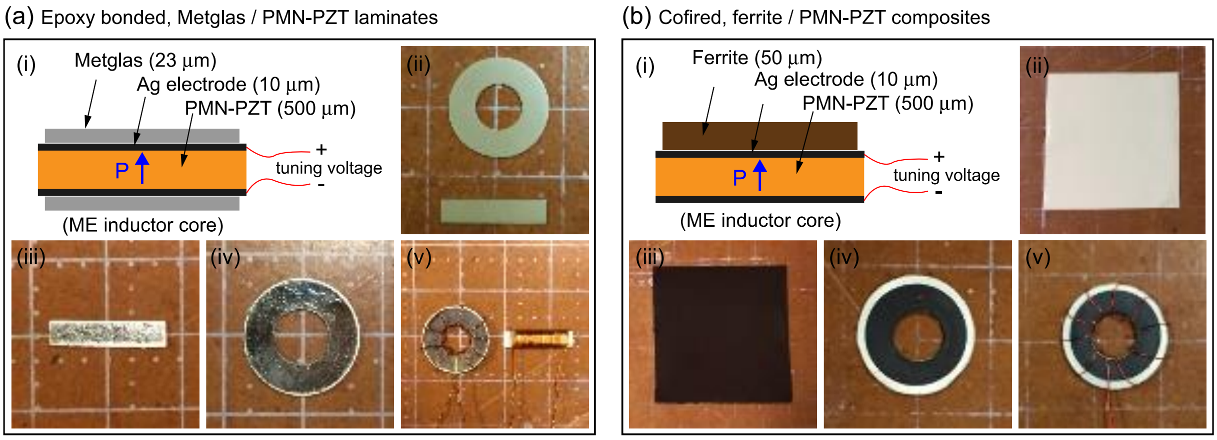

Supplementary Figure S1| Fabrication processes for VTIs. (**a**) Epoxy bonded Metglas / PMN-PZT inductor structure: (i) schematic of ME inductor core, pictures of (ii) piezoelectric PMN-PZT ceramic ring and plate, (iii) Metglas / PMN-PZT plate-type inductor core, (iv) Metglas / PMN-PZT ring-type inductor core and (v) the toroidal and solenoid inductors. (**b**) Low-temperature co-fired ferrite / PMN-PZT inductor structure: (i) schematic of inductor core, pictures of (ii) green (unfired) PMN-PZT piezoelectric ceramic tape, (iii) green NiZnCu-ferrite magnetic ceramic tape, (iv) co-fired inductor core, and (v) co-fired solenoid inductor.

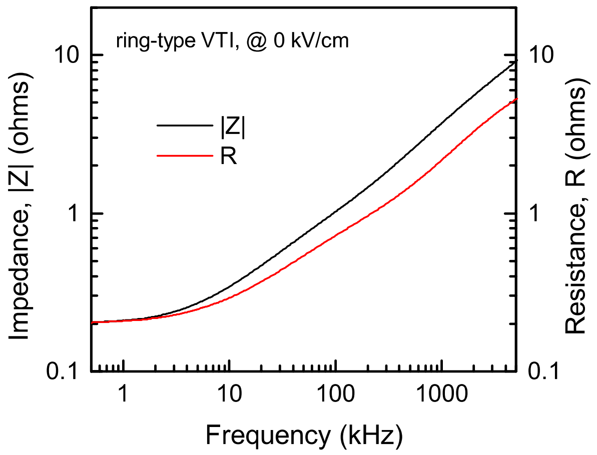

Supplementary Figure S2 | Impedance and resistance spectra of ring-type Metglas VTI at 0 kV/cm.

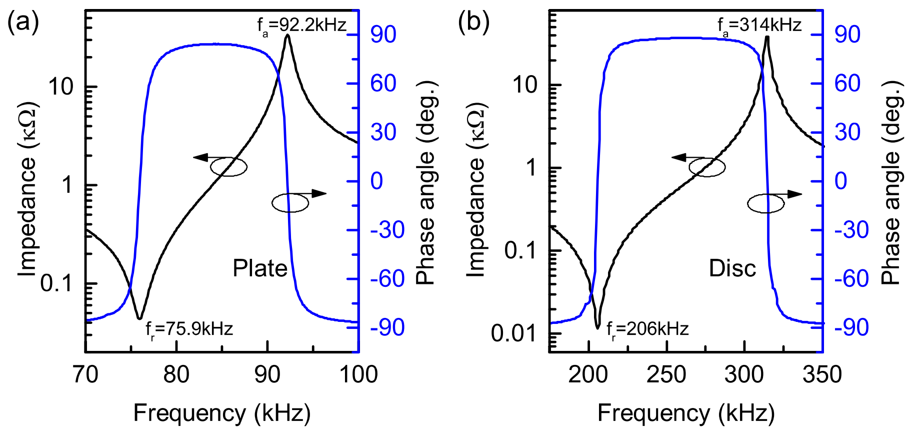

Supplementary Figure S3 | Electromechanical properties of piezoelectric ceramics. Impedance spectra of textured PMN-PZT piezoelectric ceramics: (**a**) long plate for 3-1 mode, k_{31}, and (**b**) disc for planar mode, k_p.

Supplementary Notes: Theoretical Analysis of Metglas Inductor

Consider Metglas inductor core. Since the ribbon thickness is much smaller than the inductor transverse dimensions, both plate-type and ring-type inductor cores can be modeled by the same ribbon geometry, while the difference between plate and ring is characterized by different demagnetization factors along the longitudinal direction (non-zero for plate and zero for ring). The total free energy F of the Metglas inductor core consists of contributions from magnetic anisotropy, demagnetizing field, applied stress, and magnetic field:

$$F = K_u[1 - (\mathbf{p} \cdot \mathbf{m})^2] + \frac{1}{2}\mu_0 M_s^2 (N_1 m_1^2 + N_2 m_2^2 + N_3 m_3^2) - \frac{3}{2}\lambda_s \left[\sigma_{11}\left(m_1^2 - \frac{1}{3}\right) + \sigma_{22}\left(m_2^2 - \frac{1}{3}\right)\right] - \mu_0 M_s \mathbf{H} \cdot \mathbf{m} \tag{S1}$$

where μ_0 is the permeability of free space, $\mathbf{m}=\mathbf{M}/M_s$ is a unit vector along the direction of magnetization vector $\mathbf{M}$, and M_s is the saturation magnetization. The first term describes the magnetic anisotropy energy, where K_u is the anisotropy constant, and the unit vector $\mathbf{p}$ defines the easy axis of magnetization (annealed Metglas 2605SA1 usually exhibits uniaxial anisotropy). The second term describes the demagnetization energy, where N_i is the demagnetization factor along axis-i direction (1, 2, and 3 along length, width, and thickness, respectively). The third term describes the energy of magnetoelastic coupling, where λ_s is the saturation magnetostriction constant, and σ_{ij} are the stress components (for layered magnetoelectric inductors, the piezoelectric layer only induces in-plane biaxial stress in the magnetic layer). The last term describes the Zeeman energy in the magnetic field $\mathbf{H}$.

To apply Eq. (S1) to the voltage tunable magnetoelectric inductors, consider the easy axis of magnetization along axis-2, i.e., $\mathbf{p}$=(0,1,0). Define $K_d = \mu_0 M_s^2/2$. The demagnetization factor N_1 is determined by the inductor core geometry, i.e., $N_1 \sim 10^{-3}$ for the plate (14.8 mm × 2.8 mm × 0.023 mm) [13] and $N_1=0$ for the ring, which correspond to $K_{d1} =$

$K_d N_1$~700 J/m^3 and 0 J/m^3, respectively. In comparison to the magnetic anisotropy K_u=38 J/m^3 of Metglas, the demagnetizing field produces an important role in axis-1 direction along the plate length thus must be taken into account. Along axis-2 across the ribbon width, the formation of stripe magnetic domains effectively eliminates the demagnetizing field, thus the demagnetization factor N_2~0. Along axis-3 across the ribbon thickness, however, the demagnetization factor N_3 cannot be neglected because of the much smaller ribbon thickness as compared to its length and width. Nevertheless, formation of magnetic domains also significantly reduces the demagnetizing field in axis-3 direction when magnetization component $M_3 = M_s m_3$ is induced by stress. In the following analysis of the experimental data, $K_{d3} = K_d N_3$ is treated as a fitting parameter, which depends on the detailed domain microstructures.

Taking into account above relevant material parameters, the free energy in Eq. (S1) for a Metglas inductor in external magnetic field applied along axis-1 direction is:

$$F = K_u[1 - m_2^2] + K_{d1} m_1^2 + K_{d3} m_3^2 - \frac{3}{2}\lambda_s\left[\sigma_{11}\left(m_1^2 - \frac{1}{3}\right) + \sigma_{22}\left(m_2^2 - \frac{1}{3}\right)\right] - \mu_0 M_s H m_1 \tag{S2}$$

First, consider the "ground state" at $H = 0$. Assume in-plane biaxial compressive stress $\sigma_{11} = \sigma_{22} = -\sigma$. Then Eq. (S2) becomes:

$$F = (K_u + K_{d1})m_1^2 + (K_u + K_{d3} - K_\sigma)m_3^2 + K_\sigma/3 \tag{S3}$$

where $K_\sigma = 3\lambda_s\sigma/2$, and $m_1^2 + m_2^2 + m_3^2 = 1$ has been used to eliminate m_2. Since $K_u + K_{d1} > 0$ for both plate and ring, the "ground state" with minimum energy is always reached at $m_1 = 0$, as expected from the absence of applied magnetic field. Then, Eq. (S3) is reduced to:

$$F = K m_3^2 + K_\sigma/3 \tag{S4}$$

where $K = K_u + K_{d3} - K_\sigma$ is an effective magnetic anisotropy constant. When $K > 0$ (Regime I), the "ground state" corresponds to $m_3 = 0$, thus $\mathbf{m} = (0,1,0)$ and the magnetization vector $\mathbf{M}$ lies along the material easy axis (i.e., axis-2). When $K < 0$ (Regime II), the "ground state" corresponds to $m_3 = 1$ (or equivalently $m_3 = -1$), thus $\mathbf{m} = (0,0,1)$ and $\mathbf{M}$ lies along the stress-induced easy axis (i.e., axis-3). The magnetoelectric inductors in the two "ground states" exhibit different voltage tunability behaviors, as discussed in the following Regime I and II. A transition between these two regimes occurs at $K = 0$, or $K_\sigma = K_u + K_{d3}$, which determines the critical stress for such a transition:

$$\sigma_c = \frac{2(K_u + K_{d3})}{3\lambda_s} \tag{S5}$$

In Regime II ($\sigma > \sigma_c$), the susceptibility of the magnetoelectric inductors strongly depends on the stress. Under magnetic field H applied along axis-1, Eq. (S3) becomes:

$$F_{II} = (K_{d1} - K_{d3} + K_\sigma)m_1^2 - \mu_0 M_s H m_1 + K_u + K_{d3} - 2K_\sigma/3 \tag{S6}$$

where $m_3^2 = 1 - m_1^2$ has been used since $m_2 = 0$ in this regime. The magnetic field-induced magnetization $M_1 = M_s m_1$ is obtained from $dF_{II}/dm_1 = 0$, which gives $m_1 = \mu_0 M_s H / 2(K_\sigma + K_{d1} - K_{d3})$. The magnetic susceptibility $\chi = dM_1/dH$ is given by:

$$\chi_{II} = \frac{K_d}{K_\sigma + K_{d1} - K_{d3}} \tag{S7}$$

At transition to Regime II, $K_\sigma = K_u + K_{d3}$, thus $\chi_{II} = K_d/(K_u + K_{d1})$. For a large susceptibility ($\chi \gg 1$), the tunability $\gamma = (\mu_{\sigma_c} - \mu)/\mu$ defined in terms of the permeability $\mu = 1 + \chi$ becomes essentially $\gamma = (\chi_{\sigma_c} - \chi)/\chi$, which, according to Eq. (S7), is:

$$\gamma_{II} = \frac{K_\sigma - K_u - K_{d3}}{K_u + K_{d1}} = \frac{3\lambda_s \sigma}{2(K_u + K_{d1})} - \frac{K_u + K_{d3}}{K_u + K_{d1}} \tag{S8}$$

which is a linear function of the compressive stress σ.

In Regime I ($\sigma < \sigma_c$), the susceptibility of the magnetoelectric inductors only weakly depends on the stress. In fact, if $\sigma_{11} = \sigma_{22}$ is assumed, stress would produce no effect on the permeability. The weak stress dependence comes from the small difference between σ_{11} and σ_{22}, which results from the different transverse dimensions of the layered magnetoelectric inductor structure. The magnitude of the compressive stress component in the length direction (axis-1) is expected to be slightly greater than that in the width direction (axis-2). Define $\Delta\sigma = \sigma_{22} - \sigma_{11}$, then under magnetic field H applied along axis-1, Eq. (S2) becomes:

$$F_I = (K_u + K_{d1} + K_{\Delta\sigma})m_1^2 - \mu_0 M_s H m_1 + \frac{1}{2}\lambda_s \sigma_{11} - \lambda_s \sigma_{22} \tag{S9}$$

where $K_{\Delta\sigma} = 3\lambda_s \Delta\sigma/2$, and $m_2^2 = 1 - m_1^2$ has been used since $m_3 = 0$ in this regime. The magnetic field-induced magnetization $M_1 = M_s m_1$ is obtained from $dF_I/dm_1 = 0$, which gives $m_1 = \mu_0 M_s H / 2(K_{\Delta\sigma} + K_u + K_{d1})$. The magnetic susceptibility $\chi = dM_1/dH$ is given by:

$$\chi_I = \frac{K_d}{K_{\Delta\sigma} + K_u + K_{d1}} \tag{S10}$$

At zero stress $\sigma_{11} = \sigma_{22} = 0$, $K_{\Delta\sigma} = 0$, thus $\chi_I = K_d/(K_u + K_{d1})$, which is the same as at transition to Regime II. The tunability $\gamma = (\chi_{\sigma=0} - \chi)/\chi$ is obtained from Eq. (S10):

$$\gamma_I = \frac{K_{\Delta\sigma}}{K_u + K_{d1}} = \frac{3\lambda_s \Delta\sigma}{2(K_u + K_{d1})} \tag{S11}$$

which is directly proportional to the stress component difference $\Delta\sigma$.

Next we apply the above formulated theory to analyze the experimental data of Metglas inductors. To do so, the biaxial stress σ applied to the Metglas ribbon is expressed as a function of the piezoelectric strain $\varepsilon = d_{31}E$ of the PMN-PZT layer, $\sigma = Y\varepsilon/(1-\nu) = Yd_{31}E/(1-\nu)$, where Y=110 GPa and ν=0.3 are the Young's modulus and Poisson's ratio of Metglas, d_{31}=420 pC N^{-1} is the piezoelectric constant of PMN-PZT, and E is the electric field applied to PMN-PZT layer. Consider the experimental results plotted in Fig. 2i. Regime II corresponds to the linearly fitted segments of the tunability curves at high electric field. According to Eq. (S8), the theoretical slope is:

$$\frac{d\gamma_{II}}{dE} = \frac{3\lambda_s Y d_{31}}{2(K_u + K_{d1})(1-\nu)} \tag{S12}$$

Using above material parameters and $\lambda_s = 27 \times 10^{-6}$ for Metglas into Eq. (S12) and comparing with the fitted slope of the tunability curve for ring-type inductor ($K_{d1} = 0$) shown in Fig. 2i gives $K_u = 126$ J m^{-3}, and further comparing with the fitted slope for plate-type inductor shown in Fig. 2i gives $K_{d1} = 988$ J m^{-3}. Equation (S8) also shows that the constant term (intercept) of the linear function is – $(K_u + K_{d3})/(K_u + K_{d1})$. Comparing with the fitted intercepts of the ring-type and plate-type inductors shown in Fig. 4d gives respectively $K_{d3} = 570$ J m^{-3} for the ring-type inductor and $K_{d3} = 1367$ J m^{-3} for the plate-type inductor. It is worth noting that, given the high idealization of the theoretical model formulated above, the extracted value $K_{d1} = 988$ J m^{-3} is in good agreement with the estimated value $K_{d1} \sim 700$ J m^{-3} for plate; $K_{d3} = 570$ J m^{-3} for ring and $K_{d3} = 1367$ J m^{-3} for plate are of the same order of magnitude as K_{d1}, while their difference can be attributed to different magnetic domains (and thus different demagnetization factors) in ring-type and plate-type inductors. The extracted value $K_u = 126$ J m^{-3} is about 3 times the intrinsic value $K_u = 38$ J m^{-3} for Metglas, which, nevertheless, is within the reported range of directly measured values.

Regime I corresponds to the linearly fitted initial segments of the tunability curves at low electric field. Assume $\Delta\sigma = k\sigma$. According to Eq. (S11), the theoretical slope is:

$$\frac{d\gamma_{I}}{dE} = \frac{3\lambda_s Y d_{31} k}{2(K_u + K_{d1})(1-\nu)} \tag{S13}$$

Using above material parameters into Eq. (S13) and comparing with the fitted slopes for the initial segments of the tunability curves for ring-type and plate-type inductors shown in Fig. 2i gives respectively k=0.11 for the ring-type inductor and k=0.04 for the plate-type inductor. As expected, the extracted value of k is much smaller than 1; in particular, k is negligibly small for plate-type inductor, which is due to the more symmetric geometry in the length and width directions of a plate than in the circumferential and radius directions of a ring.

Using above extracted values of the relevant parameters, the theoretical predictions of Regime I and II for both ring-type and plate-type inductors are plotted and compared with the experimental data in Fig. 5. Theoretically, the transition between Regime I and II occurs at the critical stress determined by Eq. (S5), which corresponds to the critical electric field:

$$E_c = \frac{2(K_u + K_{d3})(1-\nu)}{3\lambda_s Y d_{31}} \tag{S14}$$

Using above material parameters into Eq. (S14) gives E_c = 2.6 kV cm^{-1} for ring-type inductor and E_c = 5.6 kV cm^{-1} for plate-type inductor. These values agree well with the experimental data. It is worth noting that the experimental data exhibit a smooth transition rather than a sharp transition between the two regimes, where the tunability gradually deviates from the initial low-field linear segment of Regime I and smoothly approaches the high-field linear segment of Regime II. Such deviation from idealized theoretical model is attributed to the non-uniformities in the layered magnetoelectric inductors, such as spatial variations in K_{d3} and σ due to the microstructures of magnetic domains in Metglas ribbons and polarization domains in PMN-PZT layer.
